# Lectures on Diseases of the Skin

**Published:** 1886-07

**Authors:** Henry Wile

**Affiliations:** Lecturer on Dermatology, Atlanta, Ga.


### LECTURES ON DISEASES OF THE SKIN.

DELIVERED AT THE ATLANTA MEDICAL COLLEGE DURING THE SESSION OF 1885–6, BY HENRY WILE, M. D., LECTURER ON DERMATOLOGY, ATLANTA, GA.

### LECTURE V.

GENERAL ETIOLOGY—GENERAL ORGANIC CAUSES, IMMEDIATE, REMOTE—GENERAL LOCAL CAUSES, MECHANICAL, PARASITIC—A PROPER CLINICAL CONCEPTION.

Reported Stenographically for the ATLANTA MEDICAL AND SURGICAL JOURNAL by W. Kay Tewksbury.

In considering the subject of general etiology, reference is made to the general causes of disease. In all departments of science, especially in medicine, there has ever been a struggle to discover the cause of physical phenomena. A glance at the history of medicine is sufficient to show how meagre have been the rewards of patient investigation—how few the positive results that have appeared from time to time. Theory and speculation have never been wanting as to causes, and the opinions of medical writers of different ages oscillated like taste in the world of fashion.

In cutaneous diseases, our knowledge as to causes was extremely limited until about fifty years ago, since which time notable discoveries have been made. Besides many valuable generalizations, we have to-day a solid etiological basis for a class of diseases that are denominated *parasitic.*

In olden times most all diseases of the skin were regarded as symptomatic, and certain poisons circulating in the blood and juices of the system were in general looked upon as the causes. But modern research has exposed the fallacy of this doctrine. The causes are necessarily of a great variety and number, both on account of the exposed position of the skin and its apparently intimate, though to a great extent obscure, relations to the rest of the system and to special organs. Therefore, in discussing the general etiology of diseases of the skin, it is convenient to group the causes as follows:

1. *General organic causes.*
2. *General local causes.*

1. General organic causes are such as give rise to cutaneous disease along with other more or less profound organic disturbance. Diseases resulting thus are to be regarded as direct or primary results of causes affecting the entire organism or special organs. These causes may be immediate or remote.

Immediate causes operate with more or less rapidity, are pronounced, and stand in conspicuous or decided relation with their effects. Under this group are embraced those that give rise to the specific eruptions of variola, scarlatina, rubeola, roseola, varacella, typhoid and typhus fever, cerebro-spinal meningitis, syphilis, purpura, leprosy, rheumatism, gout. There are many diseases whose symptoms and course of development seem to be of such an independent character that some authorities are inclined to regard their pathology as entirely local. Thus diseases, like certain forms of eczema, acne, acne rosacea, urticaria and hyperidrosis, present, as a rule, local symptoms. Yet not infrequently will these processes be accompanied by other systemic disturbances, which, when closely studied, reveal some causal relations. These causal relations, when recognized, frequently indicate the proper therapeusis. Thus far we are unable to offer any physiological explanation of them. They are involved in obscurity, and as yet the evidence of their existence is purely clinical. The association of disorders of digestion with certain skin affections is a well-known clinical fact. Dyspepsia, constipation, and in general all forms of gastric and intestinal irritation, are frequently encountered in cases of acne, urticaria, eczema and rosacea. Diseases of the liver may be accompanied by jaundice and pruritus; Bright's disease of the kidneys by pruritus and œdema; diabetes by furunculosis, pruritus and gangrene; valvular heart disease by cyanosis and œdema; uterine and ovarian disorders by chloasma, eczema, acne, acne rosacea. Bulkley claims that when the last-named eruption exists to a great extent on the chin, it "is almost a sure sign of menstrual disorder." Furthermore, pruritus, local or general, herpes gestationis or eczema may co-exist with a gravid condition of the uterus and disappear usually at child-birth. In the male, acne in different forms may be seen where there has been sexual abuse, and some writers recently claim to have observed the existence of causal relations between acne and urethral disease, especially stricture.

The relation of the nervous system to the skin in its normal and pathological conditions, is an intimate one, and in some diseases positive pathological data can be given. The vaso-motor system controls the cutaneous vascular supply, and any influence whereby this supply is increased or diminished may be sufficient to lead to nutritive changes in the skin (hypertrophy or atrophy) or to special lesions. The vaso-motor system is under the direction of so-called vaso-motor centres situated in the medulla oblongata and scattered throughout the spinal cord. These centres may be irritated directly or reflexly—directly by poisonous agents, as belladonna and quinine, etc., circulating in the blood, and coming into direct contact with the centres ; reflexly by external applications of turpentine, mustard, arnica, etc., or internal use of remedies like copaiba, that may provoke a characteristic cutaneous eruption by exciting irritation of the mucous membrane of alimentary canal. But the most common and best recognized influence of the vaso-motor nerves is in the hyperæmias of the skin, as in the erythemata, and in the processes of inflammation. The rationale of the action of the vaso-motor nerves in these morbid processes has already been considered.

Then again there are a number of diseases known as *trophoneuroses*, on account of a supposed causal relations existing between the cutaneous diseases and so-called *trophic* nerves, whose centres, according to Charcot, are situated in the spinal cord, and whose irritation is followed by a disturbance of nutrition that gives rise, as a rule, to degenerative changes. Thus bed-sores and certain chronic ulcerations on the soles of the feet have been found to be associated with a sclerotic condition of the gray matter and degeneration of the ganglionic cells of the cord. There are quite a number of diseases that are regarded as *trophoneuroses*; indeed there seems to be a disposition on the part of some writers to relegate diseases with obscure pathology to this class. But it is a matter of theory and the enumeration of all these diseases is not essential.

The most striking example of the causal relation of the nervous system with disorders of the skin is seen in herpes zoster, where the cutaneous nerves of the part affected and certain spinal ganglia present a well-marked pathological condition. Nerve lesions have also been observed in connection with leprosy and pemphigus. Irritation of the sensory filaments gives rise to pruritus, and the scratching of the patient, in the effort to relieve the itching, may be so severe as to be the immediate cause of dermatitis or some forms of eczema. As immediate causes may be regarded certain articles of food, as shell-fish, strawberries, etc., which may give rise to urticaria; also many drugs used in medicine, whose ingestion may be followed by a characteristic eruption known as *dermatitis medicamentosa*. Among these drugs belladonna, balsam copaiba, quinine, bromides, iodides, arsenic, morphia and chloral may be mentioned.

As remote causes may be regarded those that have an indirect relation to their effects, that are more or less obscure, and exist only potentially. As one of the most operative of these remote factors, *heredity* must be mentioned. There can be no question as to the fact that physical and mental peculiarities are transmitted from one generation to another, and as to the transmission of disease, it is well established for syphilis. The weight of clinical evidence also argues strongly for the hereditary nature of psoriasis, leprosy, alopecia, cancer, ichthyosis, eczema, naevus and pigment anomalies. A low degree of vitality, conditions of systematic exhaustion from disease, poor living, bad hygienic surroundings form predisposing causes for such disorders as ecthyma, furnuculosis, impetigo contagiosa and chronic ulcerations. The age and sex of the individual may also be regarded as remote causes. Thus the age of infancy predisposes to seborrhœa, eczema rubrum and intertrigo, urticaria; the age of puberty to acne; old age to pruritus and new formations, especially epithelioma. The female sex is predisposed to chloasma, lupus erythematosus, seborrhœa and pruritus, while sycosis (barber's itch) is confined to the male sex.

As other minor remote conditions that have more or less influence in producing disease, the seasons, climate and occupation remain to be considered. During the cold season it is noticeable that psoriasis, ichthyosis and desquamative processes in general, also pruritus hiemalis are provoked or aggravated. Scarlet fever and variola occur more frequently in winter, while miliaria (prickly heat) occurs for the most part during the summer months. Climate, atmospheric conditions and mode of life seem to have some influence on certain diseases. It is well known that leprosy is confined to certain parts of the earth, especially the Orient, certain parts of Norway and the Sandwich Islands; prurigo and lupus vulgaris to southern Europe; elephantiasis Arabum to North Africa, especially Egypt, Arabia, Asia Minor and the West Indies; pellagra to North Italy and Spain.

Occupation may be a cause of cutaneous disease, especially in those individuals in whom there exists any predisposition to such disease. Thus workers in dye-stuffs, bleaching agents, paraffine, tar, phosphorus, etc., may be affected by eczema, or acne and chronic forms of dermatitis. Bakers, firemen and washwomen are liable to special forms of acute or chronic eczema. As a remote cause of disease, a word may be said in reference to that condition of lowered cutaneous vitality which renders the skin less able to withstand irritations of any kind. The skin forms, as it were, a *locus minoris resistentiæ*, and seems to respond to irritation more readily than any other part of the organism. Thus parasitic diseases seem to flourish on some individuals more than on others; poison oak, poison ivy, sumach, etc., excite dermatitis (venenata) on some and not on others.

2. General local causes are those that operate from without and give rise to eruptions that are peculiar to the skin. On account of the relation of the skin to the external world, there is necessarily an exposure to a great variety of irritants. Among these may be mentioned those of a mechanical character. Thus direct violence, as a blow, causes a contusion or inflammatory changes resulting in atrophy, hypertrophy or cicatrization. Long-continued pressure from tight shoes on the feet, or the hammer in the palm of the blacksmith's hand, causes hypertrophy or thickening of the epidermal layers (corn, callositas). Then again rough clothing, as flannels worn next to the skin, may prove irritating. One of the most fruitful causes of disease, especially dermatitis and eczema, is the mechanical act of scratching on the part of the individual to relieve sensory nerve irritation. Local applications of a medicinal character, such as mustard, croton oil, arnica, turpentine, carbolic acid, iodine, mineral acids, potassa, etc., are all agents that are irritating to a greater or lesser extent, and excite disease, especially eczema, when there exists that low degree of cutaneous vitality already referred to, in which the power to withstand irritation is greatly impaired. The skin may respond at once, the nature and severity of the process depending upon the character and degree of irritation. The first of these agents, including iodine, cause, as a rule, hyperæmia or inflammation with or without suppuration; the remainder are essentially destructive in their effects, and are generally followed by a necrobiosis, or death of the tissue. Extreme high or low temperature is also destructive, according to the degree of temperature change, provoking simple hyperæmia or causing complete destruction of the part exposed. Neglect and want of cleanliness, especially when there is any unusual glandular activity of the skin, are often direct exciting causes. Thus corpulent persons may suffer from intertrigo and hyperidrosis.

As local causes there remain yet to be mentioned those that provoke a special and important class of diseases, called from their etiology *parasitic.* The parasites may be vegetal or animal and the varieties have already received our attention. This class of diseases is considered as contagious, but, inasmuch as the parasites, especially the vegetal, do not flourish readily on every person on account of the non-existence of the proper conditions, the degree of contagion is variable. This point will be further developed subsequently.

Of late a parasitic origin is claimed by some pathologists for variola, scarlet fever, malignant pustule, typhoid fever, erysipelas, leprosy, lupus vulgaris, syphilis, tuberculosis, but the claims that are thus made rest, for the most part, upon the mere demonstration of the presence of micro-organisms. 'Tis true in two instances the differentiation of the parasite and its constant association with the diseased process has been confirmed as in leprosy the *bacillus lepræ* of Neisser and in malignant pustule (anthrax) the *bacillus anthracis* of Koch. As for the others the matter is still *sub judice.*

It may be well at this point to refer to the necessity of always forming a proper clinical conception of a cutaneous eruption when one is presented to your observation. Your attention has hitherto been directed to the study of the normal structure and physiological relations of the integument, and to the development of the various lesions as set forth in the general symptomatology. The general pathological processes that underlie the formation of cutaneous disease, with some of their histological details, have been discussed, and you have just now listened to a narration of the prominent causes and influences that operate to bring about these morbid conditions. In forming a clinical conception of a disease of the skin, it is of the utmost importance to keep in mind and make a proper application of all these general considerations. By comparative study you will learn that each disease of the skin possesses distinguishing characteristics, having its causes, its own manner of coming into existence, its own course of development, duration, termination and results. Therefore, to rightly interpret the manifestations of a cutaneous disease, all its peculiar symptomatic, pathological and causal relations must be recognized. A recognition of these leads to a correct clinical conception.

